# Compact SPICE Model of Memristor with Barrier Modulated Considering Short- and Long-Term Memory Characteristics by IGZO Oxygen Content

**DOI:** 10.3390/mi13101630

**Published:** 2022-09-28

**Authors:** Donguk Kim, Hee Jun Lee, Tae Jun Yang, Woo Sik Choi, Changwook Kim, Sung-Jin Choi, Jong-Ho Bae, Dong Myong Kim, Sungjun Kim, Dae Hwan Kim

**Affiliations:** 1 School of Electrical Engineering, Kookmin University, Seoul 02707, Korea; 2 Division of Electronics and Electrical Engineering, Dongguk University, Seoul 04620, Korea

**Keywords:** neuromorphic system, synaptic device, compact modeling, indium gallium zinc oxide

## Abstract

This paper introduces a compact SPICE model of a two-terminal memory with a Pd/Ti/IGZO/p^+^-Si structure. In this paper, short- and long-term components are systematically separated and applied in each model. Such separations are conducted by the applied bias and oxygen flow rate (OFR) during indium gallium zinc oxide (IGZO) deposition. The short- and long-term components in the potentiation and depression curves are modeled by considering the process (OFR of IGZO) and bias conditions. The compact SPICE model with the physical mechanism of SiO_2_ modulation is introduced, which can be useful for optimizing the specification of memristor devices.

## 1. Introduction

The Internet of Things (IoT) refers to a technology in which sensors are attached to objects to exchange data in real time over the Internet. Edge computing is used to reduce latency and bandwidth by performing computing at or near the physical location of a user or data source for IoT [1,2,3,4,5,6]. Due to the increasing variety of image sensing data, the demand for technology using IoT is increasing, and data collection and analysis technology must be specified. Memristor can be a building block in neuromorphic systems by controlling conductance [7,8,9,10,11,12,13]. Various resistive switching behaviors are observed in a lot of materials, such as oxides, nitrides and other materials [14,15,16,17,18,19,20,21,22]. Among them, the indium gallium zinc oxide (IGZO)-based memristor is very promising for memory applications because of its interface-type switching and good compatibility with conventional Si processing [14,15,16,17,18,19].

In previous studies, IGZO-based memristors have shown good synaptic properties for neuromorphic systems and memory applications [14,15,16,17,18,19,20,21]. Similarly to other oxide memristors, when Ag is used as the top electrode (TE) in IGZO-based memristors, Ag plays a dominant role in switching through the formation of the Ag filament [22,23,24]. Conversely, intrinsic resistive switching was observed when non-chemical metallization memory-type electrodes were used [25]. Here, in most cases, a gradual change in resistance is observed, indicating that the interface of the IGZO and electrode layers plays an important role in the resistance change. Non-filamentary switching, as observed in several material systems, provides better conductance control for neuromorphic system [26,27,28].

Basically, good long-term memory retention is beneficial for the neuromorphic system to ensure good performance without data loss [29,30]. Meanwhile, short-term memory with resistance drift is rather suitable for temporal learning which could save the leaning cost in reservoir computing [28]. Therefore, the distinction between the short- and long-term components in the memristor could be important. However, in previous studies, the classification of short- and long-term components has rarely been reported [31]. In our previous papers, we characterized the resistive switching and synaptic characteristics of a IGZO/SiO_2_/Si structure that is compatible with CMOS [14,15,16,17,18]. This paper introduces the compact SPICE model of a two-terminal memory with a Pd/Ti/IGZO/p^+^-Si structure in which the Si bottom electrode (BE) is favorable to a CMOS process [32,33]. The short- and long-term components are systematically separated and analyzed in each model. We proceeded with the separation of the short- and long-term components by applying bias to the samples with different oxygen flow rates (OFRs) during IGZO deposition. The compact SPICE model introduced in this paper can be used to optimize the specifications of the memristor.

## 2. Experimental Section

An initial cleaning of the p-type Si wafer with a boron doping concentration of 2 × 10^19^ cm^−3^ was conducted for the Pd/IGZO/SiO_2_/p^+^-Si memristors. The p-type Si wafer acts as the BE of the memristor system. A 60 nm-thick IGZO film was deposited at a power of 150 W using a radio-frequency (RF) sputtering system. The Ar flow was 3 sccm and the O_2_ flow rates (OFR) were 1, 1.5 and 2 sccm for the three samples, respectively. A SiO_2_ layer was formed as a native oxide between the p^+^-Si and IGZO layers [15]. Then, a 2 nm-thick Ti layer and a 40 nm-thick Pd layer were deposited by e-beam evaporation as an adhesion layer and a top electrode (TE). In addition, Ti and Pd patterning was carried out using a shadow mask with a rectangular pattern measuring 100 μm × 400 μm.

## 3. Results and Discussion

**Figure 1:**
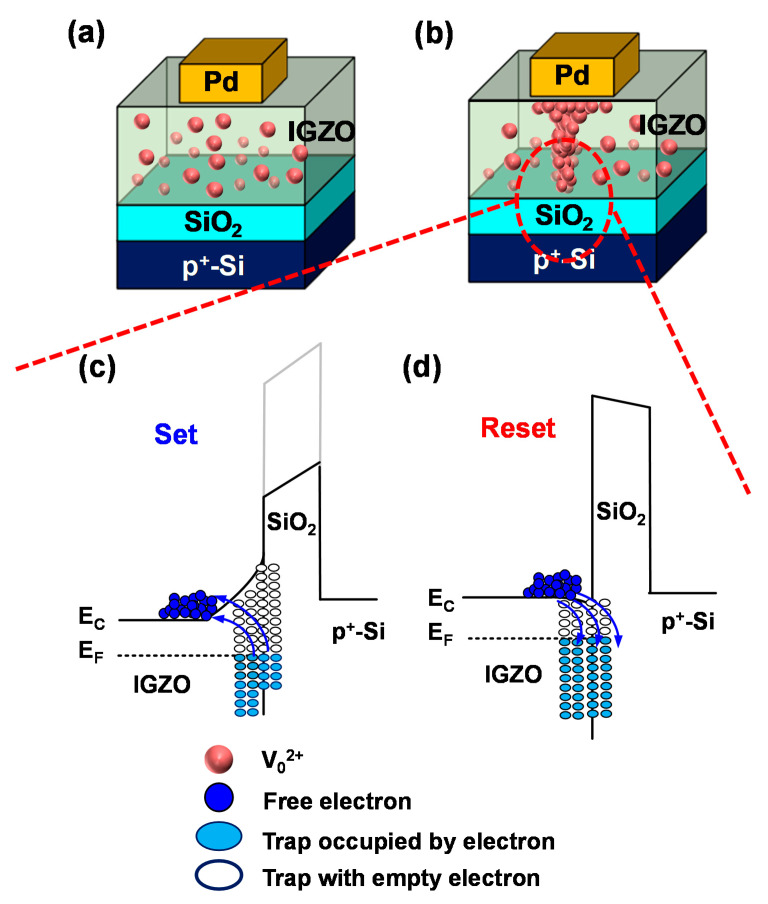
Device stacks of Pd/IGZO/SiO_2_/p^+^-Si memristors for (**a**) HRS and (**b**) LRS. Energy band diagram of the Pd/IGZO/SiO_2_/p^+^-Si memristors for (**c**) HRS and (**d**) LRS.

The typical characteristics of the memristor used in this work are as follows. When a strong negative voltage is applied to the TE, oxygen vacancies (V_0_^2+^) inside the IGZO film are driven toward the TE and the filament composed of V_0_^2+^ is formed, which extends from the TE to the BE for the formation process, as shown in Figure 1a,b. After the formation process, the Schottky barrier on the TE side collapses, and the barrier formed by the SiO_2_ layer on the BE side is modulated, resulting in the memristor switching characteristics [15,34]. The barrier height (BH) of SiO_2_ is modulated by electron trapping/detrapping in the trap at the interface between the IGZO and SiO_2_ layers. When a positive voltage is applied to the TE, the electrons are detrapped in the trap at the IGZO–SiO_2_ interface, causing a relatively positive charge. The BH is lowered and the memristor resistance decreases for the SET process in Figure 1c. Conversely, when a negative voltage is applied to TE, electrons are detrapped in the trap at the IGZO and SiO_2_ interface, resulting in a relatively negative charge, and the BH increases, thereby increasing the resistance of the memristor for the RESET process shown in Figure 1d.

The main current mechanism of the memristor is the thermionic emission of the barrier formed by native oxide [15,18,31]. The current formula by thermionic emission is the same as Equation (1):

(1) $$I=AA^{*}T^{2}\exp\left[\frac{q}{kT}\left(\sqrt{\frac{qV_{TE}}{4L_{eff}\pi \varepsilon_{SiO2}}}-\Phi \left(t\right)\right)\right]$$

where *A* is the device area; A* is the Richardson constant; *T* is the absolute temperature; *k* is the Boltzmann constant; *V_TE_* is the voltage applied to *TE*; *q* is the electric charge; *ε_SiO_*_2_ is the dielectric constant at SiO_2_; *L_eff_* is the native oxide effective thickness; and Φ(*t*) is the native oxide effective BH [12]. The barrier modulation by the memristor switching operation drops from the initial BH (Φ*_init_*(*t*)) to the BH change amount (ΔΦ(*t*)) in Equation (2).

(2) $$\Phi \left(t\right)=\Phi_{init}-\Delta \Phi \left(t\right)$$

ΔΦ(*t*) is calculated by Equation (3) for potentiation and Equation (4) for depression [12].

(3) $$\Delta \Phi \left(t\right)=\Delta \Phi_{S0}\left[1-\exp\left[-{\left(\frac{t}{\tau_{pS}}\right)}^{\beta_{pS}}\right]\right]+\Delta \Phi_{L0}\left[1-\exp\left[-{\left(\frac{t}{\tau_{pL}}\right)}^{\beta_{pL}}\right]\right]$$

(4) $$\Delta \Phi \left(t\right)=\Delta \Phi_{S}(t)\left[\exp\left[-{\left(\frac{t}{\tau_{dS}}\right)}^{\beta_{dS}}\right]\right]+\Delta \Phi_{o}(t)\left[\exp\left[-{\left(\frac{t}{\tau_{dL}}\right)}^{\beta_{dL}}\right]\right]$$

(5) $$\Delta \Phi_{L0}=\exp\left(\alpha_{VL}V_{TE}+\alpha_{OL}\mathrm{OFR}+\alpha_{L}\right)$$

(6) $$\Delta \Phi_{S0}=\exp\left(\alpha_{VS}V_{TE}+\alpha_{OS}\mathrm{OFR}+\alpha_{S}\right)$$

(7) $$\tau_{pL}=\exp\left(\gamma_{pVL}V_{TE}+\gamma_{pOL}\mathrm{OFR}+\gamma_{pL}\right)$$

(8) $$\tau_{dL}=\exp\left(\gamma_{dVL}V_{TE}+\gamma_{dOL}\mathrm{OFR}+\gamma_{dL}\right)$$

(9) $$\beta_{pL}=\kappa_{pL}\times \mathrm{OFR}+\kappa_{L}$$

Equations (3) and (4) are double-stretched exponential functions (DSEFs) composed of two sums of stretched exponential functions, and each stretched exponential function (SEF) is responsible for the modulation of the barrier during short- and long-term operations. ΔΦ*_S_*(*t*) and ΔΦ*_L_*(*t*) are the total amount of change of the current BH by the short- and long-term components, respectively.

ΔΦ*_S_*_0_, ΔΦ*_L_*_0_, *τ_pS_*, *τ_pL_*, *τ_dS_*, *τ_dL_*, *β_pS_*, *β_pL_*, *β_dS_* and *β_dL_* are all fitting parameters that form the DSEF [12]. ΔΦ*_S_*_0_, ΔΦ*_L_*_0,_ *τ_pL_*, *τ_dL_* and *β_pL_* are expressed as functions of the externally applied bias and OFR. The parameter extraction process will be described later.

**Figure 2:**
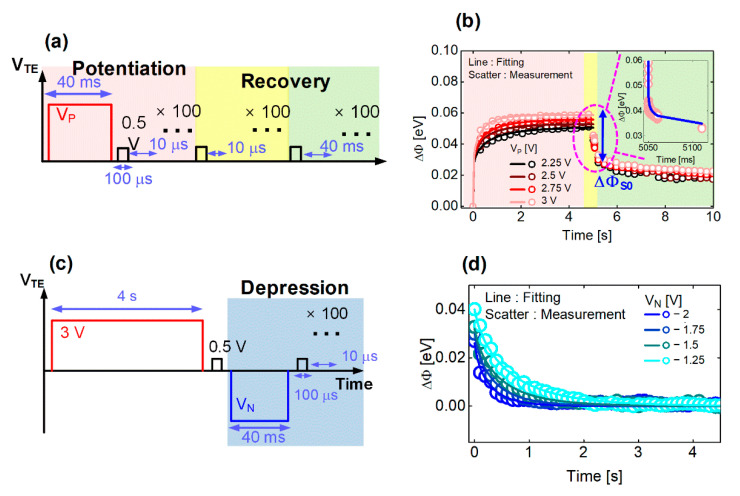
Potentiation and recovery pulse sequence to extract the DSEF modeling parameters. (**a**) Schematic of the potentiation and recovery pulse sequence (**b**) Potentiation and recovery curves when applying different V_p_: the red, yellow and green regions indicate potentiation, short-term component and long-term component of the recovery phase, respectively. (**c**) Pulse sequences to extract the long-term SEF parameter. (**d**) Extraction of *τ_dL_* and *β_dL_* using the long-term component fitting line.

To extract the parameters that constitute the DSEF from Equation (3), an experimental sequence was designed as shown in Figure 2a. Conduct the potentiation and recovery experiments simultaneously. Separate the ΔΦ*_S_* by fitting from the recovery test result. Fit the ΔΦ*_L_* component from the potentiation test result. If the above steps are performed for each potentiation voltage (V_P_), the DSEF parameter can be expressed as a function of V_P_ for the device with 2 sccm.

First, a stress pulse of magnitude V_P_ was applied to TE for 40 ms, and then a read pulse was applied for 100 μs with an interval time of 10 μs. The current was measured while applying the read pulse to observe the change in the memristor conductance at every potentiation pulse. The read operation period was 120 μs in total, including the state in which no pulse was applied. It was very short considering that the potentiation pulse width was 40 ms. The reason for this design was that the read process is performed for the shortest time possible so as not to lose the data of the short-term component during the read operation. According to the above procedure, the potentiation and read pulses are applied continuously, and this is repeated 100 times to end the potentiation phase in the red box region of Figure 2a. Once the potentiation phase is completed, we move to the recovery phase, which consists of two phases: the yellow and green regions in Figure 2a. The yellow box was designed with a tight read pulse interval of 10 μs to observe the recovery by the short-term component.

In Figure 2a, the green box is designed with a read pulse interval of 40 ms duration to see the recovery by the long-term component. In each recovery phase, 100 read pulses are applied.

The above experiment was repeated by dividing it into four cases of V_P_ = 2.25, 2.5, 2.75 and 3 V.

Figure 2b shows the results measured by the procedure proposed in Figure 2a. The current measured while applying the read pulse was converted to Φ by Equation (1), and ΔΦ was calculated through Equation (2). The results of the potentiation phase are expressed in the red region, and the results of the short- and long-term recovery phases are expressed in the yellow region and the green box, respectively.

First, as indicated by the blue arrow in Figure 2b, the decrease in ΔΦ with a steep slope was regarded as the barrier change due to the short-term component that was defined as ΔΦ*_S_*_0_. ΔΦ*_S_*_0_ is extracted for each V_P_ and can be expressed as a bias function (Equation (6)). Second, *τ_pS_* and *β_pS_* are extracted by fitting short-term components, as shown in the inset of Figure 2b. Here, two assumptions are made about the short-term component. The first assumption is that *τ_dS_* = *τ_pS_* and *β_dS_* = *β_pS_*. The second assumption is that *τ_dS_* and *β_dS_* are independent of bias and OFR. The change in BH of the short-term component proceeds at least 200 times faster than that of the long-term component. Therefore, it is very difficult to detect the *τ* and *β* trend of the short-term component by the bias or process conditions; therefore, the above assumption was made. Third, ΔΦ*_L_*_0_, *τ_pL_*, and *β_pL_* are extracted by fitting while fixing ΔΦ*_S_*_0_, *τ_pS_* and *β_pS_* in the red region of Figure 2b with Equation (3). Repeating for each V_P_, ΔΦ*_L_*_0_ (Equation (5)), *τ_pL_* (Equation (7)) and *β_pL_* can be expressed as a function of bias.

Then, we designed an experimental sequence as shown in Figure 2c to extract the long-term SEF parameter of Equation (4). First, we applied the potentiation pulse of 3 V to the TE for 4 s to sufficiently increase the conductance of the memristor, and then applied a depression pulse. For the depression phase, as shown in the blue box region in Figure 2c, a stress pulse with a magnitude of V_N_ is applied for 40 ms, and then a read pulse is applied for 100 μs with an interval time of 10 μs. The depression and read pulses are continuously applied and this is repeated 100 times.

The result of the above experimental procedure is shown in Figure 2d, and as shown in Figure 2c, the current measured in the read pulse was converted to ΔΦ by Equations (1) and (2). The long-term SEF parameter extraction procedure of Equation (4) is as follows. *τ_dL_* and *β_dL_* are extracted by fitting (line) the measurement data (scatter) as shown in Figure 2d, with the long-term part of Equation (4). By fitting the entire V_N_, the parameters *τ_dL_* (Equation (8)) and *β_dL_* can be expressed as a function of the bias.

**Figure 3:**
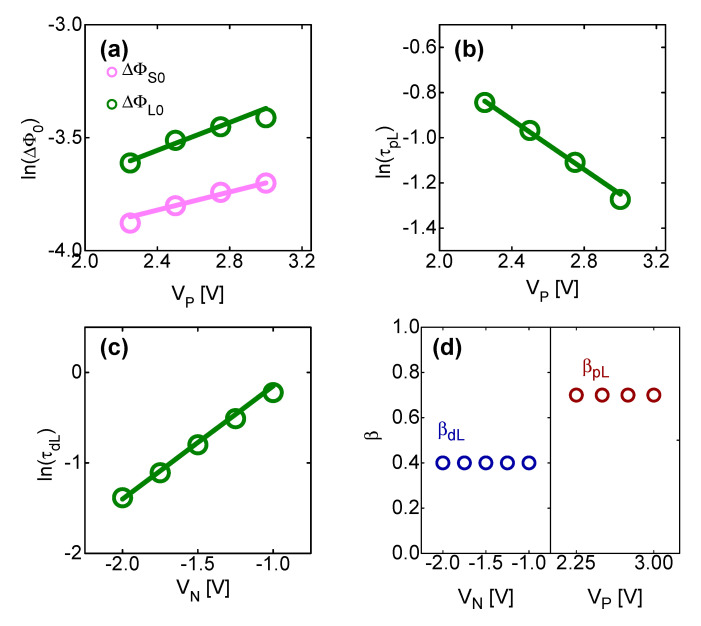
(**a**) ΔΦ*_S_*_0_ and ΔΦ*_L_*_0_ as a function of V_P_; (**b**) *τ_pL_* as a function of V_p_; and (**c**) *τ_dL_* as a function of V_N_. (**d**) *β_pL_* and *β_dL_* as a function of V_N_ and V_P_, respectively.

Figure 3a shows the experimentally revealed relationship between V_P_, ΔΦ*_S_*_0_ and ΔΦ*_L_*_0_.

ΔΦ*_S_*_0_ and ΔΦ*_L_*_0_ are the BH of the short- and long-term components that can be shifted to the maximum when *V_TE_* is V_P_. It was empirically verified that both ΔΦ*_S_*_0_ and ΔΦ*_L_*_0_ increase exponentially with respect to V_P_. Conversely, Figure 3b shows the experimentally revealed relationship between *τ_pL_* and V_P_. *τ_pL_* is a parameter related to the detrapping time of electrons by the long-term component in the potentiation. It was empirically found that Φ*_pL_* decreases exponentially with V_P_. The higher the voltage applied to TE, the lower the Fermi level at the SiO_2_–IGZO interface, so the concentration of traps where electrons can be detrapped increases. Therefore, the maximum changeable BH (ΔΦ*_S_*_0_ and ΔΦ*_L_*_0_) increases. Conversely, the probability that V_P_ can cause electron detrapping at the SiO_2_–IGZO interface increases, so *τ_pL_* (detrapping time) decreases.

Figure 3c shows the experimentally revealed relationship between V_N_ and *τ_dL_*. *τ_dL_* is a parameter related to the electron trapping time by the long-term component in the depression. *τ_dL_* was empirically found to increase exponentially with V_N_. As the voltage applied to the TE increases, the fermi level at the SiO_2_ and IGZO interface decreases, so the concentration of trap sites that can trap electrons decreases. Therefore, the time for trapping electrons increases.

Figure 3d shows the relationship between *β_pL_*, *β_dL_*, V_P_ and V_N_. *β_pL_* and *β_dL_* are parameters that indicate the rate of change over time of *τ_pL_* and *τ_dL_* by long-term components during potentiation and depression, respectively. It was experimentally found that *β_pL_* and *β_dL_* are independent of the bias applied to the TE.

**Figure 4:**
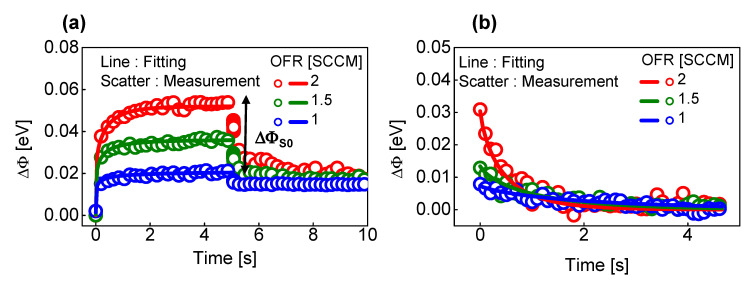
(**a**) Measurement and fitting results of ΔΦ in different OFRs (1, 1.5 and 2 sccm) when applying the pulse sequence of Figure 2a with a V_P_ of 2.5 V; and (**b**) Measurement and fitting results of ΔΦ in different OFRs (1, 1.5 and 2 sccm) when applying the pulse sequence of Figure 2c with a V_N_ of −1.5 V.

Then, when the parameter extraction process in Figure 2 proceeds for the sample with different OFRs, the DSEF parameter (Equations (3) and (4)) can be expressed as a function of the OFRs. Figure 4 shows the results of the experiments conducted for this purpose. Figure 4a shows the experiment results when the 2.5 V V_P_ is fixed independently of the OFR in the pulse sequence of Figure 2a. The DSEF parameters (ΔΦ*_S_*_0_, ΔΦ*_L_*_0_, *τ_pL_* and *β_pL_*) of Equation (3) were extracted for the different OFRs, and the extraction procedure is the same as that of the method shown in Figure 2b. Similarly, Figure 4b shows the experimental results when V_N_ is −1.5 V regardless of the OFR in the pulse sequence of Figure 2c. The long-term SEF parameters (*τ_dL_* and *β_dL_*) of Equation (4) were extracted for the samples with different OFRs by Figure 4b, and the extraction procedure is the same as that in Figure 2d.

**Figure 5:**
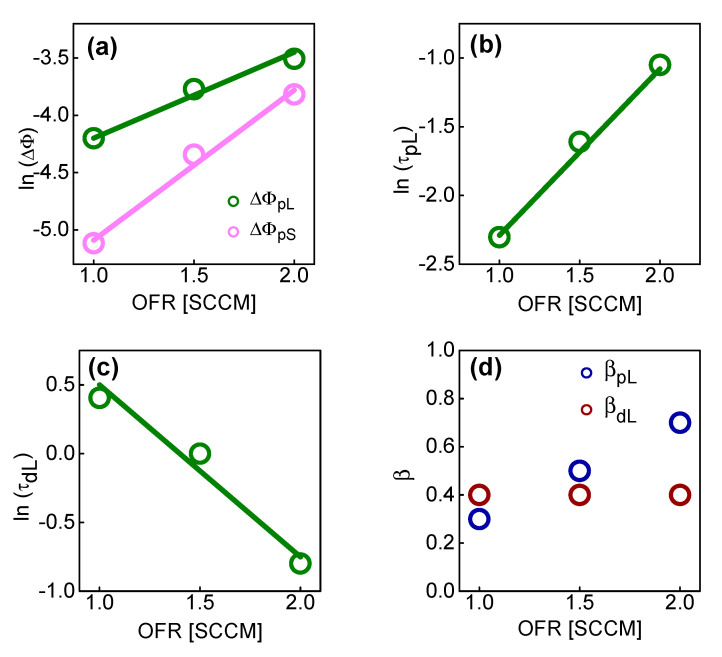
(**a**) ΔΦ_pL_ and ΔΦ_pS_ as a function of OFR; (**b**) *τ_pL_* as a function of OFR; and (**c**) *τ_dL_* as a function of OFR. (**d**) *β_pL_* and *β_dL_* as a function of OFR.

Figure 5 shows the plots of the parameters with different OFRs for ΔΦ_S0_, ΔΦ*_L_*_0_, *τ_pL_*, *τ_dL_*, *β_pL_*, and *β_dL_* extracted from the measurement data of Figure 4. Figure 5a shows ΔΦ*_S_*_0_ and ΔΦ*_L_*_0_ as a function of OFR. Figure 5b,c shows *τ_pL_* and *τ_dL_* as a function of OFR. Figure 5d shows *β_pL_* and *β_dL_* as a function of OFR.

It was experimentally found that ΔΦ*_S_*_0_, ΔΦ*_L_*_0_ and *τ_pL_* increase exponentially with increasing OFR, but *τ_pL_* decreases exponentially as OFR increases. As OFR increases, the traps between IGZO and SiO_2_ increase. There are two main reasons for this trend. The first is that the roughness of the IGZO–SiO_2_ interface increases and the trap concentration increases. This could be associated with the short-term component. The second is that, when the OFR increases, the bulk trap inside the IGZO increases because the oxygen peroxide inside the IGZO breaks bonds and acts as an electron trap. This could be related to the long-term component. The long-term switching mechanism could be caused by the charge change due to the reaction between oxygen peroxide and excess oxygen of the BE side IGZO. The excess oxygen is converted into oxygen peroxide for the SET process. Conversely, the oxygen peroxide in converted back into excess oxygen for the RESET process. Therefore, as OFR increases, the trap concentration near SiO_2_ increases; therefore, ΔΦ*_S_*_0_ and ΔΦ*_L_*_0_ increase.

During the potentiation operation, the resistance of the switching site in the SiO_2_ layer gradually decreases, so the actual voltage applied to SiO_2_ gradually decreases. This phenomenon has the effect of lowering *τ* by rapidly saturating the conductance change of the memristor. The lower the OFR, the more severe the phenomenon in which the voltage applied to the switching site gradually decreases during the potentiation operation. Therefore, the lower the OFR, the lower the *τ*.

Conversely, for the depression operation, the resistance of the switching site gradually increases, so the voltage actually applied to the SiO_2_ gradually increases. This has the effect of increasing *τ* as it can sustain the change in the conductance of the memristor. The lower the OFR, the higher the voltage applied to the switching place increases during the depression operation, which has the effect of increasing *τ*. Therefore, the lower the OFR, the higher the *τ*.

Although *β_pL_* increases with increasing OFR, *β_dL_* remains constant, however, the cause remains to be discussed further. Therefore, finally, by combining the results of Figure 3 and Figure 5, the correlation between the parameters (including ΔΦ*_S_*_0_, ΔΦ*_L_*_0_, *τ_pL_* and *τ_dL_*), the bias and OFR can be found. Among them, ΔΦ*_S_*_0_, ΔΦ*_L_*_0_, *τ_pL_* and *τ_dL_* are expressed in Equations (5)–(9).

Table 1 includes the initial parameters extracted by OFR. *L_eff_* is the effective SiO_2_ thickness that determines the thermionic emission. When a voltage is actually applied to the TE, the voltage is distributed between the IGZO and the SiO_2_, however, the current model is designed so that all voltages are applied to the SiO_2_. Therefore, the actual *L_eff_* of SiO_2_ is determined by the degree of voltage distribution between the IGZO and SiO_2_, and the model is designed so that all voltages are applied to SiO_2_ with a thickness of *L_eff_*. *L_eff_* decreases as the resistance of the IGZO decreases and that of SiO_2_ increases. The larger the OFR, the thicker the SiO_2_ (as the SiO_2_ resistance increases), and so the *L_eff_* decreases. Since the concentration of V_O_^2+^ in the IGZO increases as the OFR decreases, the IGZO becomes positively charged as a whole and the initial BH decreases.

From the results of Figure 3 and Figure 5, the parameters *α_VL_*, *α_OL_*_,_ *α_L_*, *α_VS_*, *α_OS_*, *α_S_*, *γ_pVL_*, *γ_pOL_*, *γ_pL_*, *γ_dVL_*, *γ_dOL_*, *γ_dL_*, *κ_pL_* and *κ* can be determined (Table 2, lines 1–4). In addition, the parameters independent of OFR and bias are constant (Table 2, lines 5–8).

**Figure 6:**
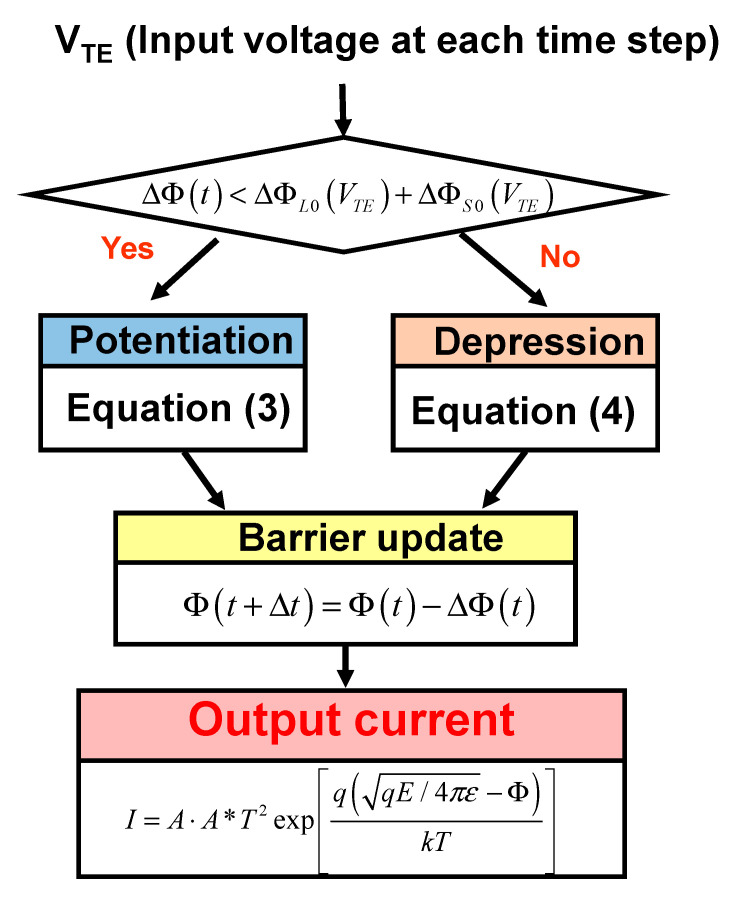
Algorithm of the compact model designed with SPICE’s Verilog-A for the barrier-modulated memristor.

Figure 6 shows the algorithm of the compact memristor model designed with SPICE’s Verilog-A. The simulation proceeds in a time-transient manner, and Figure 6 schematically shows the calculated algorithm in one time step.

The input is the voltage and the output is the current composed by the thermionic emission formula [11]. Additionally, the size of BH is updated at each time step. The maximum value that BH can be modulated by the input voltage is (Φ*_L_*_0_(*V_TE_*) + Φ*_S_*_0_(*V_TE_*)). If (Φ*_L_*_0_(V*_TE_*) + Φ*_S_*_0_(*V_TE_*)) is greater than the current change in ΔΦ(*t*), potentiation is performed. If (Φ*_L_*_0_(*V_TE_*) + Φ*_S_*_0_(*V_TE_*)) is smaller than the current BH change in ΔΦ(*t*), depression is executed. This is because the BH must be lower. The potentiation is calculated by Equation (3), and the depression is calculated by Equation (4). If Φ is calculated every time step by the potentiation and depression operation, Φ is finally determined in the thermionic emission equation to calculate the current as output.

**Figure 7:**
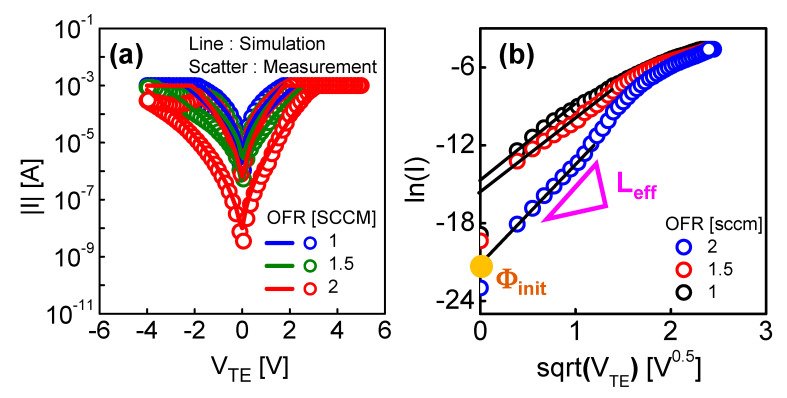
(**a**) I–V curves and (**b**) ln(I) versus *V_TE_*^1/2^ the memristor devices with different OFR conditions (1, 1.5 and 2 sccm) to extract Φ_init_.

To verify that the memristor current mechanism is thermionic emission, the I–V curve in Figure 7a is presented as a relationship between ln(I) versus *V_TE_*^1/2^, and ln(I) and *V_TE_*^1/2^ show a linear relationship which was confirmed in Figure 7b. Here, as shown in Figure 7b, $\frac{q}{kT}\cdot \sqrt{\frac{q}{4\pi \varepsilon L_{eff}}}$ can be known by the slope of the HRS current line, and *L_eff_* can be extracted from this relationship. Moreover, by the point where V = 0, we can know that $\ln\left(AA^{*}T^{2}\right)-\frac{q}{kT}$Φ*_init_*, and from it, we can extract Φ*_init_*. Figure 7a shows the I–V characteristics of memristors with different OFRs, and we compare the measurement and simulation results. The I–V curves of the memristors were applied to the TE in a double sweep in 0.1 V steps with the range of −5 V to 5 V, and the compliance current was set to 1 mA. Figure 7a shows that the measurement and simulation results are well matched.

**Figure 8:**
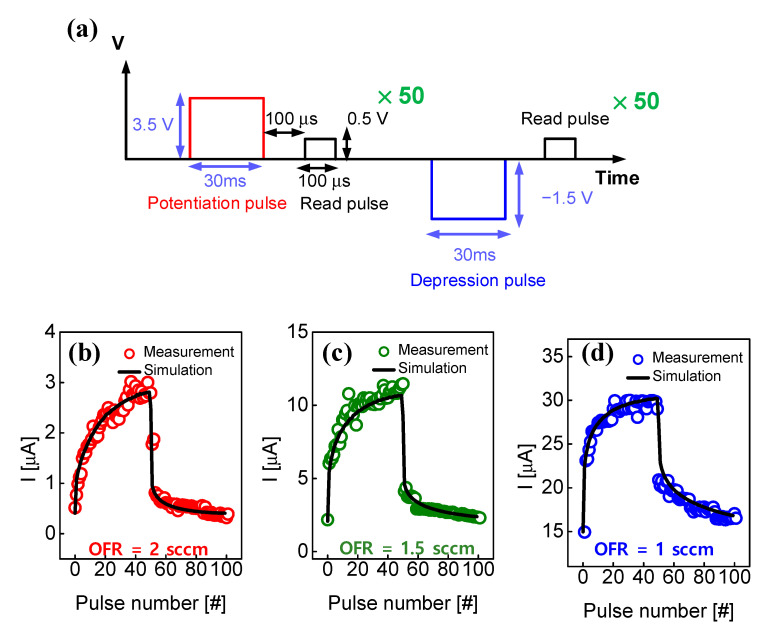
(**a**) Pulse scheme for potentiation and depression. Potentiation and depression curves of measurement and simulation as a function of pulse number for devices with different OFRs: (**b**) 2 sccm, (**c**) 1.5 sccm and (**d**) 1 sccm.

Figure 8 shows the potentiation and depression behaviors of the memristors with the different OFRs, and the experimental results and simulations were compared. The potentiation pulse had an amplitude of 3.5 V and a width of 30 ms, and a read pulse with an amplitude of 0.5 V and a width of 100 μs was applied with an interval of 100 μs. After repeating the potentiation pulse and the read pulse 50 times, a depression pulse was applied. The depression pulse was set at −1.5 V amplitude and 30 ms width, and a read pulse was applied under the same conditions as the potentiation. Similarly, the depression and read pulses were repeated 50 times and the experiment was completed. Figure 8b,c show the device results with 2 sccm and 1.5 sccm, respectively. Figure 8d shows the result of the device with 1 sccm, which has a good match. The memristor conductance was determined differently depending on the size and interval of the stimulation pulses. The stronger and longer the stimulus, the closer the memristor conductance is to long-term memory. Conversely, when the stimulus is weaker and shorter, the memristor conductance does not increase, and the conductance increases temporarily and then only decreases at the time of stimulation, acting close to short-term memory.

**Figure 9:**
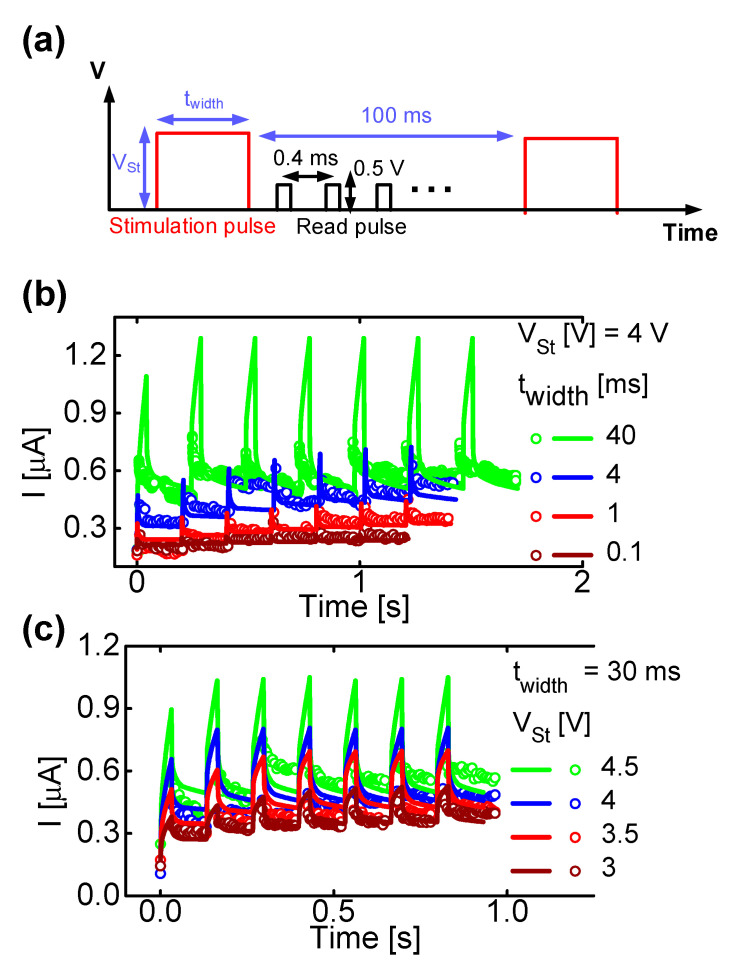
(**a**) Pulse schemes including stimulation and read pulses to emulate short- and long-term memory characteristics. (**b**) Conductance change by controlling the pulse width (t_width_) and (**c**) amplitude of the stimulated pulse (V_st_).

In addition, the memristor model introduced in this paper can reproduce short- and long-term memory behaviors depending on the stimulation conditions. To confirm this, a pulse sequence was designed as shown in Figure 9a. The stimulation pulse is used to increase the conductance of the memristor, and the interval between each stimulation pulse is fixed at 100 ms. In addition, to observe the conductance change in detail up to the short-term component, a read pulse of 0.5 V amplitude β was applied at 0.4 ms intervals. Figure 9b shows the results of dividing the stimulation pulse width (t_width_) into 0.1, 1, 4 and 40 ms while keeping the stimulation pulse amplitude (V_St_) constant at 4 V. Figure 9c shows the results of dividing the stimulation pulse width (t_width_) to 30 ms and the stimulation pulse amplitude (V_St_) to 4.5, 4, 3.5 and 3 V. As the stimulus is delivered over a longer period of time, the conductance of the memristor gradually increases and operates close to the long-term memory, and the simulation reproduces the trend well.

## 4. Conclusions

This paper introduced a compact SPICE model of a two-terminal memory with a Pd/Ti/IGZO/p^+^-Si structure. The modeling of the SiO_2_ BH modulation related to the switching operation is the core of this work. We separated the BH modulation into short- and long-term components through potentiation, depression and recovery experiments with very tight pulse intervals. The empirical model for short- and long-term memory was designed by conducting the above experiment for the device with different OFR conditions when biasing. As the magnitude of the potentiation pulse and OFR increases, the maximum value of the BH modulation increases exponentially. Conversely, as the magnitude of the potentiation pulse increases, the potentiation speed increases; however, as the OFR increases, the potentiation speed decreases. As the amplitude of the depression pulse increases and the OFR increases, the depression rate increases. We expect that this model can be useful in optimizing the memristor specifications for short- and long-term memories.

## Figures and Tables

**Table 1:** Thermionic emission current parameters.

OFR	2	1.5	1	Unit
*L_eff_*	12	25	27	nm
Φ*_int_*	0.76	0.64	0.57	eV
*A*	40,000			μm^2^
A*	32			cm^−2^ K^2^
*T*	300			K

**Table 2:** DSEFs parameters.

Parameter	Value
*α_VL_, α_OL_*, *α_L_*	0.31 V^−1^, 0.75 min/cm^−3^, −5.275
*α_VS_*, *α_OS_*, *α_S_*	0.2 V^−1^, 1.31 min/cm^−3^, −5.9
*γ_pVL_*, *γ_pOL_*, *γ_pL_*	−0.55 V^−1^, 1.21 min/cm^−3^, −2.125
*γ_dVL_*, *γ_dOL_*, *γ_Dl_*	1.25 V^−1^, −1.25 min/cm^−3^, −1.375
*κ_pL_*, *κ_L_*	0.4, −0.1
*β_dL_*	0.4
*τ_pS_* = *τ_dS_*	2 ms
*β_pS_* = *β_dS_*	0.2

## Data Availability

Not applicable.
